# Association between serum uric acid/high-density lipoprotein cholesterol ratio and hypertension among reproductive-aged women

**DOI:** 10.1186/s41043-023-00458-3

**Published:** 2023-11-08

**Authors:** Xiaoxue Han, Xuan Tan, Mengyuan Liu, Yiling Wei, Andong He, Ying Pan, Di Qiu, Ruiman Li

**Affiliations:** Department of Obstetrics and Gynecology, The First Affiliated Hospital of Jinan University, No. 613, Huangpu Road West, Tianhe District, Guangzhou, 510630 Guangdong China

**Keywords:** High-density lipoprotein cholesterol, Hypertension, Nutrition surveys, Inflammation, Women

## Abstract

**Background:**

Uric acid/high-density lipoprotein cholesterol ratio (UHR) is a novel index of inflammation and metabolism that has been investigated in various diseases. However, association between UHR and hypertension among reproductive-aged women is unclear.

**Methods:**

In this cross-sectional study, we investigated the association between serum UHR and hypertension among 5485 women aged 20–44 years based on the National Health and Nutrition Examination Survey (NHANES) database using various methods, including univariate and multivariate logistic regression analysis, stratified analysis, and spline regression. *P* < 0.05 was considered statistically significant.

**Results:**

There was significant difference in UHR between the women with and without hypertension (*P* < 0.001). After adjusting for several covariates, UHR was positively correlated with hypertension (OR > 1, *P* < 0.001). In the subgroup analysis, the positive correlations still remained between UHR and hypertension in women with various age and those with BMI ≥ 30 kg/m^2^ (*P* < 0.05) excepted for adjusting for all covariates. We further found an inflection point of the threshold effect for UHR, and the prevalence of hypertension showed different increased trends below and above the threshold.

**Conclusion:**

This study indicated a positive association between serum UHR and hypertension among reproductive-aged women, indicating that UHR is a potential clinical marker of hypertension in women.

## Background

Hypertension is a common disease that seriously endangers the human health worldwide. It is universally acknowledged that hypertension is a risk factor for cardiovascular disease [1], renal disease [2, 3], stroke [4], and vascular dementia [5]. It is reported that the prevalence of hypertension was 45.4% in 2017–2018 among adults aged 18 years and over in the USA [6]. Although its prevalence was lower among women (39.7%) than men (51.0%), hypertension in women still deserves great attention. For example, increased life stress and work-related anxiety generally affect women with hypertension more significantly than men [7]. Additionally, women with chronic hypertension have a significantly increased risk of developing preeclampsia during pregnancy, affecting the safety of the mother and fetus [8]. Although there are many drugs to treat hypertension, early prediction and diagnosis of hypertension are of great importance, especially among reproductive-aged women. Currently, several objective indicators, such as Angiotensin II and aldosterone [9], may be helpful for clinical management of hypertension, but it is necessary to continue to explore the novel biomarkers or clinical indexes for prediction or diagnosis of hypertension among reproductive-aged women.

Hypertension is characterized by chronic, low-grade inflammation and metabolic imbalance [10]. Recently, several studies indicated that uric acid/high-density lipoprotein cholesterol ratio (UHR) is a novel index of inflammation. To our knowledge, association between UHR and inflammatory diseases, such as thyroiditis [11], diabetic nephropathy [12], and non-alcoholic fatty liver disease [13], has been well established. Therefore, studying UHR in hypertension may make sense. Notably, G. Aktas et al. [14] found that the level of UHR was significantly high among the participants with poor blood pressure control. However, no previous studies have specifically investigated the association between UHR and hypertension among reproductive-aged women. Our study was designed to fill this knowledge gap. Therefore, the study was performed to investigate whether UHR is a potential index of hypertension in reproductive-aged women.

## Materials and methods

### Study design and population

The National Health and Nutrition Examination Survey (NHANES) is a research project of the National Center for Health Statistics that collects data on the health and nutritional status of the civilian, non-institutionalized population of the USA. The survey data are released every 2 years. In addition, all participants have written informed consent for data collection before any data collection [15].

**Fig. 1:**
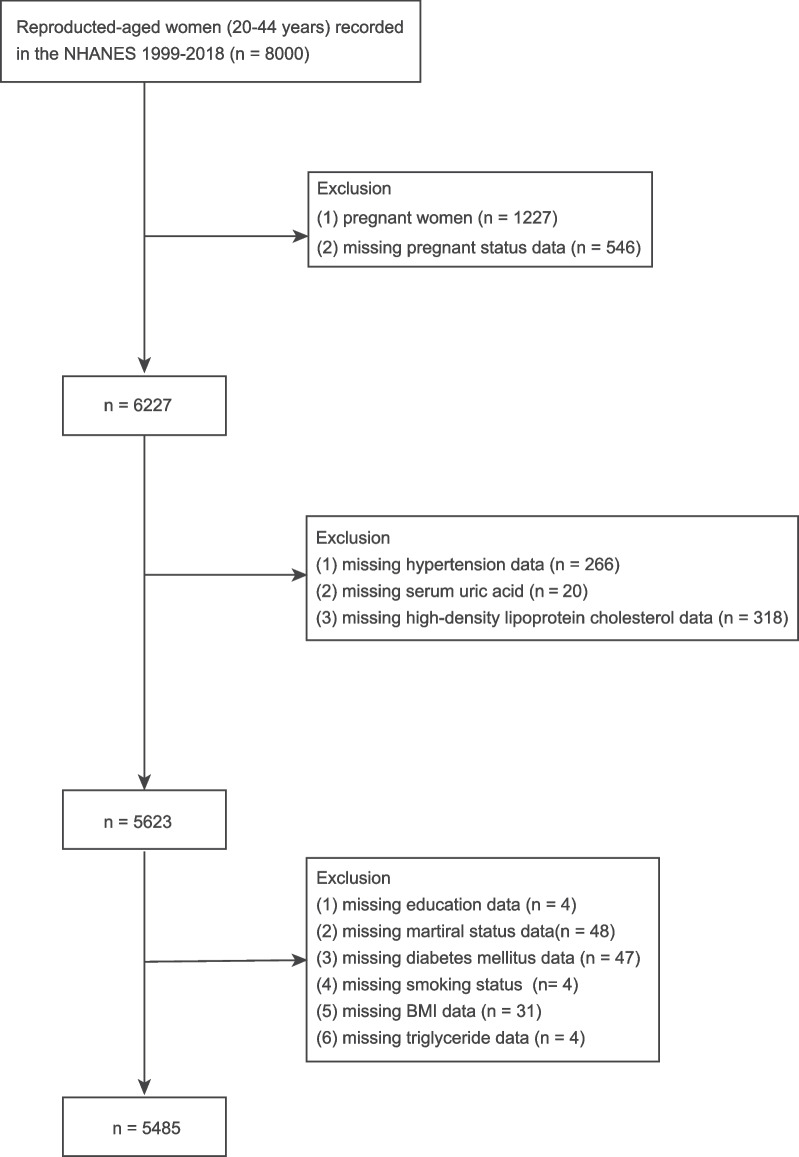
Flowchart of participant selection from the NHANES 1999–2018

A total of 8000 women aged 20–44 years were recorded in the NHANES database from 1999 to 2018. Therein, participants with the following characteristics were excluded: (1) women who were pregnant (*n* = 1227) and missing pregnancy status (*n* = 546); (2) those with missing information of hypertension (*n* = 266), serum uric acid (n = 20), and high-density lipoprotein cholesterol (*n* = 318); (3) those with missing education (*n* = 4), marital status (*n* = 48), diabetes mellitus (*n* = 47), smoking status (*n* = 4), body mass index (BMI) values (*n* = 31), and triglyceride (*n* = 4). Therefore, 5485 women were finally included in this analysis (Fig. 1).

### The exposure and outcome variables' definition

The exposure variable was the UHR, which was determined as serum uric acid divided by high-density lipoprotein cholesterol. Hypertension diagnosis was determined by a combination of self-reported physician diagnosis, use of medication for hypertension, and having a systolic blood pressure ≥ 130 or/and a diastolic blood pressure ≥ 80 mmHg according to the American Heart Association/American College of Cardiology 2017 guideline for monitoring and diagnosis of hypertension [16].

### Covariates

The covariates are demographic information, physical examinations, laboratory data, and questionnaire data. Demographic information included age, race, education level, and marital status. Laboratory data included triglycerides and total cholesterol. Physical examinations included BMI. Additionally, questionnaire data included smoking behavior and diabetes mellitus (yes or no). Therein, the smoking status was defined as current (smoked > 100 cigarettes in their lifetime and currently smoked some days or every day), past (smoked > 100 cigarettes in their lifetime but currently did not smoke at all), and never (smoked < 100 cigarettes in their lifetime) [17].

### Statistical analyses

Categorical variables were presented using percentages [*n* (%)]. For continuous variables, we first performed a normality test; those that obeyed the normal distribution were presented using mean ± standard deviation, whereas those that did not obeyed the normal distribution were presented using median and quartiles [*M* (*Q*1, *Q*3)]. We found that the UHR data are unevenly distributed and clearly skewed to the right. Therefore, prior to conducting regression analysis, the values of UHR need to be in-transformed. Univariable logistic regression analysis was used to screen covariates, and variables with statistically significant were included in multivariable logistic regression analysis. Three models were established to evaluate the association between UHR and hypertension. Model 1 was a univariable logistic regression model, model 2 was a multivariable logistic regression model adjusted for age, race, education, and marital status, and model 3 was a multivariable logistic regression model adjusted for age, race, education, marital status, diabetes mellitus, BMI, smoking status, triglycerides, and total cholesterol. The odds ratio (OR) with 95% confidence interval (CI) was used to report associations. Stratified analyses were performed based on age (< 35 years and ≥ 35 years) and BMI (< 25 kg/m^2^ and 25, < 30 kg/m^2^ and ≥ 30 kg/m^2^). Finally, a spline regression was ultimately used to assess whether there was a linear relationship between UHR and hypertension. R software and EmpowerStats were used for data analysis, and *P* < 0.05 was considered statistically significant.

## Results

### Baseline characteristics of the participants with or without hypertension

A total of 5485 reproductive-aged women were included in the final analysis. Compared to the non-hypertension group, participants in the hypertension group were more likely to be older, other Hispanic, high school education or below, widowed/divorced/separated, and with BMI ≥ 30 kg/m^2^. Significant differences were also observed between the two groups for smoking and diabetes mellitus status. Meanwhile, women with hypertension had higher level of serum uric acid, lower level of high-density lipoprotein cholesterol, and higher levels of UHR, triglycerides, and total cholesterol (Table 1).

**Table 1:** Basic characteristics of the research population with and without hypertension

	Total *n* = 5485	Non-hypertension *n* = 4819	Hypertension *n* = 666	*P* value
Age (years)	33.00 (26.00–39.00)	32.00 (25.00–39.00)	37.00 (31.00–41.00)	< 0.001
Race, *n* (%)				< 0.001
Mexican American	1185 (21.60)	1085 (22.52)	100 (15.02)	
Non-Hispanic white	453 (8.26)	407 (8.45)	46 (6.91)	
Non-Hispanic black	2355 (42.94)	2101 (43.60)	254 (38.14)	
Other Hispanic	1144 (20.86)	916 (19.01)	228 (34.23)	
Other race	348 (6.34)	310 (6.43)	38 (5.71)	
Education, *n* (%)				0.024
Less than high school	1230 (22.42)	1060 (22.00)	170 (25.53)	
High school or equivalent	1156 (21.08)	1004 (20.83)	152 (22.82)	
College or above	3099 (56.50)	2755 (57.17)	344 (51.65)	
Marital status, *n* (%)				< 0.001
Married/cohabiting	3198 (58.30)	2806 (58.23)	392 (58.86)	
Widowed/divorced/separated	697 (12.71)	575 (11.93)	122 (18.32)	
Never married	1590 (28.99)	1438 (29.84)	152 (22.82)	
BMI, *n* (%)				< 0.001
< 25 kg/m^2^	2110 (38.47)	1985 (41.19)	125 (18.77)	
≥ 25 and < 30 kg/m^2^	1458 (26.58)	1322 (27.43))	136 (20.42)	
≥ 30 kg/m^2^	1917 (34.95)	1512 (31.38)	405 (60.81)	
Diabetes mellitus, *n* (%)				< 0.001
No	5324 (97.06)	4723 (98.01)	601 (90.24)	
Yes	161 (2.94)	96 (1.99)	65 (9.76)	
Smoking status, *n* (%)				< 0.001
Current	1304 (23.77)	1090 (22.62)	214 (32.13)	
Past	633 (11.54)	541 (11.23)	92 (13.81)	
Never	3548 (64.69)	3188 (66.15)	360 (54.05)	
Uric acid (mg/dL)	4.40 (3.70–5.10)	4.30 (3.70–5.00)	4.80 (4.10–5.60)	< 0.001
High-density lipoprotein cholesterol (mg/dL)	54.00 (44.00–64.00)	54.00 (45.00–65.00)	50.00 (41.00–61.00)	< 0.001
UHR	0.08 (0.06–0.11)	0.08 (0.06–0.10)	0.10 (0.07–0.12)	< 0.001
Triglyceride (mmol/L)	1.03 (0.72–1.55)	0.99 (0.71–1.50)	1.29 (0.86–1.92)	< 0.001
Total cholesterol (mmol/L)	4.73 (4.16–5.40)	4.71 (4.16–5.38)	4.91 (4.34–5.61)	< 0.001

Data are presented as number (%) or median and quartile range

*BMI* body mass index, *UHR* serum uric acid/high-density lipoprotein cholesterol ratio

### Associations between UHR and hypertension

Firstly, univariate logistic regression analysis was performed to analyze the associations between the collected variables and hypertension (Table 2). The results showed that several variables, including age, non-Hispanic Black, other Hispanic, widowed/divorced/separated, BMI ≥ 25 kg/m^2^, with diabetes mellitus, with smoking, uric acid, UHR, triglycerides, and total cholesterol, were significantly positively associated with hypertension. Additionally, more than high school education, never married, and high-density lipoprotein cholesterol were significantly negatively associated with hypertension.

**Table 2:** Univariate logistic regression for variables associating with hypertension

	OR (95% CI)	*P* value
Age (years)	1.07 (1.06, 1.08)	< 0.001
Race, *n* (%)		
Mexican American	Ref	
Non-Hispanic white	1.23 (0.85, 1.77)	0.276
Non-Hispanic black	1.31 (1.03, 1.67)	0.028
Other Hispanic	2.70 (2.10, 3.47)	< 0.001
Other race	1.33 (0.90, 1.97)	0.156
Education, *n* (%)		
Less than high school	Ref	
High school or equivalent	0.78 (0.64, 0.95)	0.013
College or above	0.76 (0.62, 0.92)	0.006
Marital status, *n* (%)		
Married/cohabiting	Ref	
Widowed/divorced/separated	1.52 (1.22, 1.90)	< 0.001
Never married	0.76 (0.62, 0.92)	0.006
BMI, *n* (%)		
< 25 kg/m^2^	Ref	
≥ 25 and < 30 kg/m^2^	1.63 (1.27, 2.10)	< 0.001
≥ 30 kg/m^2^	4.25 (3.44, 5.25)	< 0.001
Diabetes mellitus, *n* (%)		
No	Ref	
Yes	5.32 (3.84, 7.37)	< 0.001
Smoking status, *n* (%)		
Never	Ref	
Past	1.51 (1.18, 1.93)	0.001
Current	1.74 (1.45, 2.09)	< 0.001
Uric acid (mg/dL)	1.54 (1.43, 1.66)	< 0.001
High-density lipoprotein cholesterol (mg/dL)	0.99 (0.98, 0.99)	< 0.001
UHR	3.02 (2.44, 3.73)	< 0.001
Triglyceride (mmol/L)	1.28 (1.19, 1.37)	< 0.001
Total cholesterol (mmol/L)	1.21 (1.12, 1.31)	< 0.001

*BMI* body mass index, *CI* confidence interval, *OR* odds ratio; ref, reference, *UHR* Uric acid/high-density lipoprotein cholesterol ratio

The adjusted correlation between UHR and hypertension is presented in Table 3. In the unadjusted model (model 1), we found that the UHR were positively associated with the prevalence of hypertension (OR 3.02, *P* < 0.001). After adjusting for multiple covariates, the positive correlation between UHR and hypertension remained significant in the model 2 and 3 (model 2: OR 3.24, *P* < 0.001; model 3: OR 1.77, *P* < 0.001). After converting UHR to a categorical variable (quartiles), the UHR levels of the Q4 groups were still positively correlated with the prevalence of hypertension compared with the lowest quartile of UHR (Q1). In addition, the trend remained significant among different UHR quartile groups (*P* for trends < 0.05 in the three models) (Table 3). The subgroup analyses stratified by age and BMI are reported in Table 4. For women with different ages, those with higher UHR levels both had a higher incidence of hypertension than those with lower levels. Furthermore, we found that higher UHR levels were consistently associated with the increased risk of hypertension in the group with BMI ≥ 30 kg/m^2^ excepted for the model 3.

**Table 3:** Logistic regression of UHR for the risk of hypertension

	Model 1, OR (95% CI)	Model 2, OR (95% CI)	Model 3, OR (95% CI)
UHR	3.02 (2.44, 3.73)***	3.24 (2.60, 4.02)***	1.77 (1.36, 2.31)***
UHR (quartiles)			
*Q*1	Ref	Ref	Ref
*Q*2	1.18 (0.90, 1.54)	1.20 (0.91, 1.57)	0.99 (0.74, 1.31)
*Q*3	1.54 (1.19, 2.00)***	1.68 (1.29, 2.19)***	1.12 (0.84, 1.48)
*Q*4	2.90 (2.29, 3.68)***	3.08 (2.41, 3.93)***	1.63 (1.22, 2.18)***
*P* for trend	< 0.001	< 0.001	0.001

*BMI* body mass index, *CI* confidence interval, *OR* odds ratio, *Ref* reference, *UHR* serum uric acid-to-high-density lipoprotein cholesterol ratio. Model 1: no covariates were adjusted. Model 2 was adjusted for age, race, education, and marital status. Model 3 was adjusted for age, race, education, marital status, smoking status, BMI, diabetes mellitus, triglycerides, and total cholesterol. ***P < 0.001

**Table 4:** Subgroup analysis stratified by age and BMI

	Model 1, OR (95%CI)	Model 2, OR (95%CI)	Model 3, OR (95%CI)
Age (years)			
< 35	3.35 (2.40, 4.68)***	3.49 (2.48, 4.90)***	1.60 (1.04, 2.46)*
≥ 35	2.98 (2.26, 3.92)***	2.98 (2.25, 3.95)***	1.50 (1.06, 2.12)*
BMI (kg/m^2^)			
< 25	1.27 (0.73, 2.21)	1.47 (0.84, 2.58)	2.07 (1.44, 2.98)***
≥ 25 and < 30	1.55 (0.96, 2.52)	1.94 (1.18, 3.18)**	1.59 (0.91, 2.76)
≥ 30	1.82 (1.33, 2.48)***	2.24 (1.61, 3.11)***	1.19 (0.66, 2.14)

*CI* confidence interval, *OR* odds ratio. Model 1: no covariates were adjusted; Model 2 was adjusted for age, race, education, and marital status; Model 3 was adjusted for age, race, education, marital status, smoking status, BMI, diabetes mellitus, triglycerides, and total cholesterol. *P < 0.05, **P < 0.01, ***P < 0.001

### Nonlinear results of UHR and hypertension

**Fig. 2:**
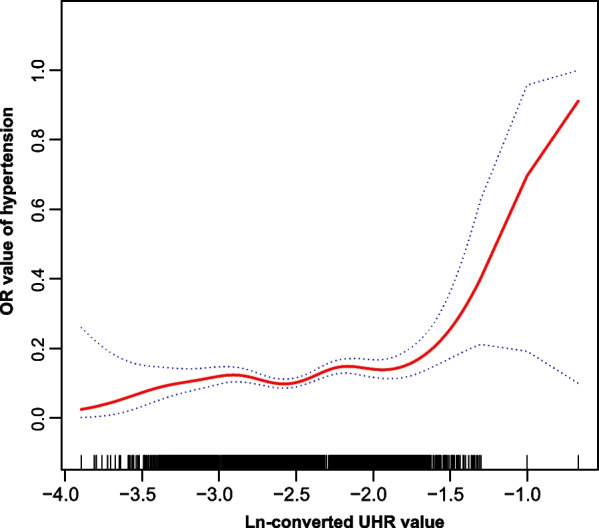
The association between UHR and hypertension. The solid red line represents the smooth curve fit between variables. Blue bands represent the 95% confidence interval from the fit

Smooth curve fitting was performed after adjusting for confounding factors in model 3, and the results indicated that the association between UHR and hypertension was nonlinear over the entire range of UHR (Fig. 2). We further found that the inflection point of the threshold effect for lnUHR was -1.86, and the prevalence of hypertension slowly increased with UHR below the threshold (OR 1.36, *P* = 0.037) and then significantly increased above the threshold (OR 8.64,* P* = 0.002) (Table 5).

**Table 5:** Nonlinearity addressed through two-piecewise linear model

	Adjusted OR (95%CI)	*P* value
Inflection point (K)	− 1.86	
lnUHR < − 1.86	1.36 (1.02, 1.83)	0.037
lnUHR ≥ − 1.86	8.64 (2.19, 34.06)	0.002
Log likelihood ratio	0.013	

*CI* confidence interval, *OR* odds ratio, *UHR* serum uric acid/high-density lipoprotein cholesterol ratio

## Discussion

Using a nationally representative sample of the reproductive-aged women in the USA, the present study obtains several major findings. First, a significant difference existed in UHR between the women with and without hypertension. Second, the univariate logistic regression analysis showed that UHR was positively correlated with hypertension, and the positive correlations remained after adjusting for several covariates. Third, UHR was also correlated with hypertension in women with different age and those with BMI ≥ 30 kg/m^2^ when adjusting for several covariates. Finally, we found an inflection point of the threshold effect for UHR and the prevalence of hypertension showed different increased trends below and above the threshold.

Inflammation, oxidative damage, and endothelial dysfunction are involved in the pathophysiology of hypertension [18, 19]. Elevated uric acid can contribute to atherosclerosis by damaging blood vessel walls, which is closely associated with hypertension, dyslipidemia, and insulin resistance [20]. High level of uric acid activates the renin-angiotensin system and reduces the synthesis of insulin-induced nitric oxide in endothelial cells [21, 22]. Additionally, dyslipidemias, including elevated level of low-density lipoprotein cholesterol and decreased level of high-density lipoprotein cholesterol, is common in patients with hypertension. Among them, high-density lipoprotein cholesterol can protect vascular endothelial cells through anti-inflammatory effects [23]. Therefore, both high uric acid and low high-density lipoprotein cholesterol may be associated with an increased risk of hypertension, and their ratio, i.e., UHR, has been widely used in various inflammatory diseases, such as poorly controlled hypertension, type 2 diabetes mellitus, metabolic syndrome, or ischemic heart disease [14, 24–26] since a single parameter may not be enough to predict or diagnose diseases. To the best of our knowledge, this is the first study to explore UHR and the risk of hypertension in reproductive-aged women. However, the current evidence only supports this association and cannot be applied to clinical prediction or diagnosis.

In this study, we found that multiple variables, such as older age and widowed/divorced/separated status, were positively correlated with hypertension, whereas educational attainment was negatively correlated with it. It is widely knowledge that older age is a risk factors of hypertension [27]. Additionally, previous studies have indicated that widowhood can increase a woman’s risk of hypertension by 92% [28]. Although the mechanisms underlying the effect of marital status on hypertension are not fully understood, previous studies have suggested that this may be due to various factors, such as neuroendocrine pathways, psychopathological factors, health behaviors, or immune pathways [29]. For example, unhappy relationships may contribute to poorer health, and marital problems often predict psychopathology, such as mood and anxiety [30]. Distressed couples exhibit greater negative affect, possibly related to cardiovascular and neuroendocrine responsive biological mediators [31], for example, greater negative behavior during marital interactions is associated with elevated catecholamine levels [32]. Additionally, previous studies have indicated that education level was negatively associated with the prevalence of hypertension among both men and women [27]. Another study also showed an association between educational attainment and better awareness of blood pressure among women [33]. High educational attainment can improve the awareness and control of hypertension. People with higher education may have better blood pressure control. Studies have found that those with higher formal education were more aware of their overall health and are more likely to receive medication, which ultimately leads to better blood pressure control [34, 35]. Notably, social determinants of health have a greater impact on the prevalence of hypertension in women than in men [36]. Therefore, we adjusted these confounding factors in logistic regression models and found that UHR level is still positively correlated with hypertension among reproductive-aged women.

There are several limitations in this study, such as the relatively small sample size. Besides, this is a cross-sectional study so we cannot infer a causal relationship between UHR and hypertension, and we do not know yet whether it can predict the onset of hypertension in advance. In order to promote the clinical application of UHR in hypertension, we should validate these correlations in women populations in other regions and explore whether elevated UHR levels could predict hypertension, including its sensitivity and specificity, through prospective cohort studies. In addition, whether we can combine UHR with other indicators to predict hypertension, as well as the timing of UHR measurement, are all questions that need to be answered before clinical translation. Further experiments will help to understand the underlying mechanisms behind the link between UHR and hypertension.

## Conclusion

Serum UHR was independently and positively correlated with the prevalence of hypertension among reproductive-aged women. After converting UHR to quartiles, the Q4 of UHR was also positively correlated with hypertension. In the subgroup analysis, this association remained positive in various age stages as well as in the women with BMI ≥ 30 kg/m^2^ (except for the model 3). Furthermore, an inflection point of the threshold effect for lnUHR in the smooth curve fitting was found to be -1.86, and the prevalence of hypertension slowly increased with UHR below the threshold and then significantly increased above the threshold. However, further studies are warranted to validate these associations and to elucidate the mechanisms underlying these associations between serum UHR and hypertension.

## Data Availability

The data of this study are publicly available on the NHANES (http://www.cdc.gov/nchs/nhanes/).

## Abbreviations

BMI: Body mass index
CI: Confidence interval
NHANES: National Health and Nutrition Examination Survey
OR: Odds ratio
UHR: Uric acid/high-density lipoprotein cholesterol ratio
